# Characterization of different-sized human αA-crystallin homomers and implications to Asp151 isomerization

**DOI:** 10.1371/journal.pone.0306856

**Published:** 2024-07-11

**Authors:** Jiayue Sun, Toshiya Matsubara, Tamaki Koide, Kirsten J. Lampi, Larry L. David, Takumi Takata

**Affiliations:** 1 Department of Chemistry, Graduate School of Science, Kyoto University, Sakyo-ku, Kyoto, Japan; 2 Shimazu Corporation, Nakagyo-ku, Kyoto, Japan; 3 Rexxam Corporation, Chuo-ku, Osaka-shi, Osaka, Japan; 4 Oregon Health and Science University, Integrative Biosciences, Portland, Oregon, United States of America; 5 Institute for Integrated Radiation and Nuclear Science, Kyoto University, Kumatori-cho, Sennan-gun, Osaka, Japan; Durham University, UNITED KINGDOM OF GREAT BRITAIN AND NORTHERN IRELAND

## Abstract

Site-specific modifications of aspartate residues spontaneously occur in crystallin, the major protein in the lens. One of the primary modification sites is Asp151 in αA-crystallin. Isomerization and racemization alter the crystallin backbone structure, reducing its stability by inducing abnormal crystallin–crystallin interactions and ultimately leading to the insolubilization of crystallin complexes. These changes are considered significant factors in the formation of senile cataracts. However, the mechanisms driving spontaneous isomerization and racemization have not been experimentally demonstrated. In this study, we generated αA-crystallins with different homo-oligomeric sizes and/or containing an asparagine residue at position 151, which is more prone to isomerization and racemization. We characterized their structure, hydrophobicity, chaperone-like function, and heat stability, and examined their propensity for isomerization and racemization. The results show that the two differently sized αA-crystallin variants possessed similar secondary structures but exhibited different chaperone-like functions depending on their oligomeric sizes. The rate of isomerization and racemization of Asp151, as assessed by the deamidation of Asn151, was also found to depend on the oligomeric sizes of αA-crystallin. The predominant isomerization product via deamidation of Asn151 in the different-sized αA-crystallin variants was L-β-Asp *in vitro*, while various modifications occurred around Asp151 *in vivo*. The disparity between the findings of this *in vitro* study and *in vivo* studies suggests that the isomerization of Asp151 *in vivo* may be more complex than what occurs *in vitro*.

## Introduction

Lens tissue is initially colorless, with its clarity maintained by highly concentrated soluble proteins, over 90% of which are crystallins. These proteins form various-sized homo- and hetero-oligomeric structures through subunit–subunit interactions, which are crucial for maintaining visual acuity throughout a lifetime [1, 2]. Different-sized crystallin oligomers play essential roles in the lens, and numerous studies have explored their sizes and structures [3–7]. However, replicating the high concentrations (300~500 mg/mL) of crystallin aggregates *in vitro* that perfectly mimic the eye’s lens is technically challenging. The oligomeric size of crystallins may also be linked to spontaneous amino acid modifications in the protein sequence. Additionally, lens fiber cells lack turnover functions, resulting in the accumulation of post-translational modifications in crystallins as they age [8]. These modified crystallin structures may lead to aggregation through abnormal subunit–subunit interactions, ultimately contributing to the formation of senile cataract by creating a relatively large-sized insoluble fraction in aging eyes [9–14].

**Fig 1:**
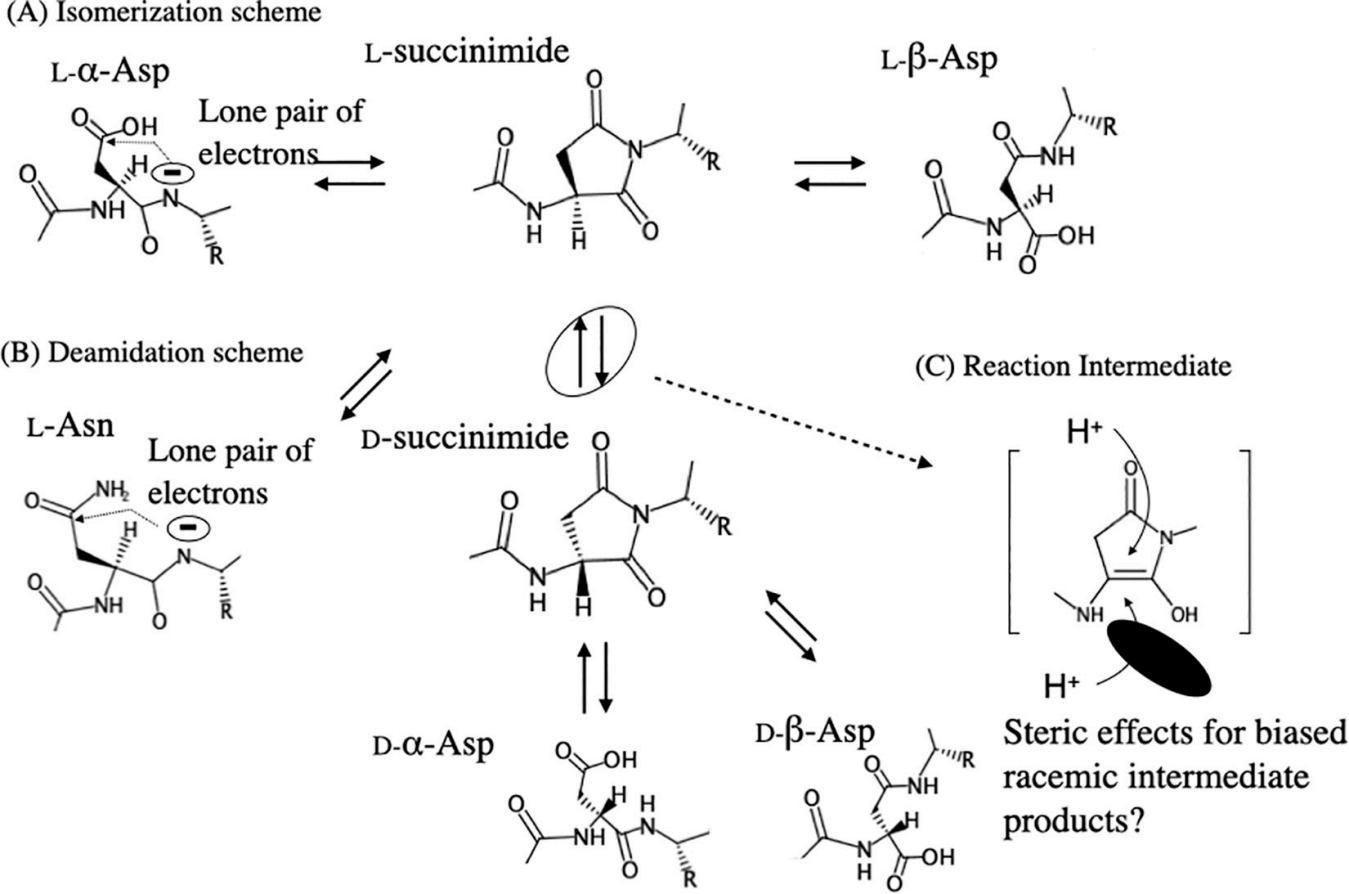
Scheme for isomerization, racemization, and deamidation of Asp/Asn. This figure outlines the hypothesized steric effects of protein tertiary structure on reaction intermediates: (A) Isomerization and racemization processes. (B) Deamidation process.

Proteomic research has indicated that the predominant modifications of lens crystallins are the non-enzymatic deamidation of asparagine residues (Asn) and isomerization/racemization of aspartate residues (Asp) [14–19]. These modifications occur spontaneously and site-specifically under physiological conditions, proceeding through the formation of a five-membered-ring L-succinimide intermediate, an enol-type intermediate, and a D-succinimide intermediate, as depicted in Fig 1. The initial event is triggered by a nucleophilic attack by the nitrogen atom of Asp/Asn on the adjacent carbonyl group. This is followed by the formation of a racemized five-membered-ring succinimide intermediate through hydrogen detachment and reattachment, producing L-/D-conformations. The succinimide ring is then opened to form Lα-/Lβ-/Dα-/Dβ-Asp, determining the ratio of isomerized Asp.

This modification is also identified in creatine kinase B and histone H2B in the mammalian brain, which is associated with a loss of enzymatic activity [20, 21]. However, the role of this modification in the lens remains elusive.

A previous study of a model peptide described how environmental factors such as buffer pH and temperature influence the deamidation/isomerization process of Asp/Asn [22]. For instance, certain sequences in crystallins favor the production of the reaction intermediate by applying less steric hindrance and promote Asp/Asn isomerization (Fig 1). This kind of steric hindrance originates from long-distance intra-subunit and inter-subunit interactions. Robinson et al. examined the contributions from the short-range steric effects of the N-terminal and C-terminal neighboring residues for deamidation of Asn/Glutamine (Gln) to Asp/Glutamate (Glu) [23]. They reported that the formation of imide intermediates of Asn/Gln is more likely when the amino acid at the C-terminal side is less bulky. In fact, many Asp sites comply with this rule; at sites where the neighboring residue of Asp/Asn is small, such as Glycine, Alanine, or Serine, Asp/Asn isomerization is more likely to occur [24–27].

Conversely, the long-range steric effects such as inter-subunit interaction on Asp/Asn isomerization/racemization *in vivo* remain obscure, primarily because it is challenging to produce different-sized recombinant crystallins containing sites that can be easily modified even *in vitro*. To investigate the long-range effects of protein assembly on Asp/Asn isomerization/racemization, a model protein containing amino acids that can be rapidly isomerized/racemized under physiological conditions is necessary. In this regard, we recently reported that isomerization via deamidation of Asn, which substituted Asp151 of αA-crystallin (D151N), proceeded much faster compared to isomerization of Asp151 without mutation [28]. Thus, the D151N variant protein is a useful tool to screen factors promoting post-translational modifications of αA-crystallin. Additionally, it has also been reported that the oligomerization of αA-crystallin varies depending on the N-terminal sequences of αA-crystallin [4, 29]. We used the N-terminal tag system to generate different-sized αA-crystallin variants. The biochemical properties of each variant and their contributions towards Asp/Asn isomerization were analyzed to observe the size contribution to the isomerization at Asp/Asn151 of αA-crystallin.

## Materials and methods

### Chemicals

Tris (hydroxymethyl) aminomethane, sodium chloride, TFA, and HPLC solvents were purchased from Sigma-Aldrich (St. Louis, MO, USA). Modified trypsin/Lys-C mix was purchased from Promega (Madison, WI, USA). TCEP was obtained from Thermo Fisher Scientific (CA, USA). Other chemicals were sourced from FUJIFILM Wako Pure Chemical Corporations (Osaka, Japan).

### Preparation of recombinant human lens αA-crystallins

Expression and purification of recombinant human αA-crystallin (αA) and αAD151N were performed as previously described [30]. The expression vectors for his-SUMO fusion αA-crystallin, his-SUMO fusion βB1-crystallin, and his-ulp-1 protease were kindly provided by Dr. Larry David (Oregon Health & Science University, Portland, Oregon). Site-directed mutagenesis at Asp151 of his-SUMO fusion αA was carried out using the QuikChange Mutagenesis kit (Stratagene, La Jolla, CA, USA); the forward primer was CAGACTGGCCTGgctGCCACCCACGCC (mutated codon in lower case). The D151N mutation of his-SUMO fusion αA was confirmed by DNA sequencing (Eurofin Genomics, Tokyo, Japan). αA and αAD151N variants were purified as previously detailed [28]. His-SUMO fusion αA, his-SUMO fusion αAD151N, and his-SUMO fusion βB1-crystallin were purified using a cobalt spin column equilibrated in 50 mM sodium phosphate buffer (pH 7.4), 10 mM imidazole, and 0.3 M NaCl, followed by elution with 250 mM imidazole, according to the manufacturer’s manual (Thermo Fisher Scientific, CA, USA). All his-SUMO-fusion proteins were incubated with ulp-1 protease at 4°C overnight, and then his-SUMO tags were removed using a new cobalt spin column (termed “cleaved-αA,” “cleaved-αAD151N,” and “cleaved-βB1”). The eluted fractions were collected, concentrated via Amicon ultrafiltration (Millipore, Billerica, MA, USA), and dialyzed against 50 mM sodium phosphate buffer (pH 7.0). The purity (> 98%) of the recombinantly expressed proteins, with tags completely removed, was confirmed by SDS-PAGE. Additionally, αA-crystallin variants were digested into peptides by trypsin, and the mutation was verified by mass spectrometry (LTQ; Thermo Finnigan, San Jose, CA, USA). Protein concentration in all experiments was estimated by measuring the intrinsic UV absorbance of each solution at 280 nm.

### Analytical size-exclusion chromatography

Each αA-crystallin variant (2.0 mg/mL) was filtered through a 0.45 μm membrane and then applied to a Superose 6 Increase column (Cytiva, Tokyo, Japan) equilibrated in 20 mM Tris-HCl buffer (pH 7.8) containing 150 mM NaCl. Samples were eluted at a flow rate of 0.25 mL/min and monitored at 280 nm. The molar mass of each αA-crystallin variant was calculated using the following molecular weight protein standards: bovine thyroglobulin (669 kDa), horse spleen ferritin (type-1: 440 kDa), aldolase (158 kDa), bovine serum albumin (67 kDa), chicken ovalbumin (43 kDa), chymotrypsinogen A (25 kDa), and bovine pancreatic ribonuclease A (13.7 kDa). Data represents the mean ± standard deviation of three individual experiments.

### Circular dichroism

Circular dichroism measurements in the Far-UV range (Far-UVCD) were conducted using a J-805 spectropolarimeter (JASCO, Tokyo, Japan), as previously detailed [31]. Briefly, 0.2 mg/mL of each αA-crystallin variant was prepared in 50 mM sodium phosphate buffer (pH 7.0) in a cell with a 0.02 cm path length. All spectra were recorded at 25°C, with the buffer spectrum subtracted from the sample spectra.

### Native fluorescence and bis-ANS measurements

The sole tryptophan residue (Trp9) in the N-terminal extension of the αA-crystallin subunit was utilized to examine the Trp environment at the N-terminus of each αA-crystallin variant [29, 31]. A concentration of 0.1 mg/mL for each αA-crystallin variant was prepared in 20 mM Tris-HCl buffer (pH 7.8) containing 150 mM NaCl, and fluorescence spectra were measured using a 2 mL sample volume cuvette in an FP-8250 fluorescence spectrometer (JASCO, Tokyo, Japan). The excitation wavelength was set at 290 nm, and emission spectra were recorded from 300 to 400 nm. To assess the hydrophobic surface of each αA-crystallin variant, fluorescence of bis-ANS mixed with each variant was measured. Briefly, 0.01 mg/mL of bis-ANS was mixed with 0.1 mg/mL of each αA-crystallin variant in 20 mM Tris-HCl buffer (pH 7.8) containing 150 mM NaCl. The mixed samples were placed in a 0.1 mL sample volume cuvette in the same fluorescence spectrometer. The excitation wavelength was set at 390 nm, and emission spectra were recorded between 400 and 600 nm.

### Chaperone assay

The chaperone-like activity of each αA-crystallin variant was evaluated by measuring their ability to prevent reducing-induced precipitation of insulin and heat-induced precipitation of cleaved-βB1. Initially, 0.1 mg/mL of each αA-crystallin variant was incubated with 0.4 mg/mL of insulin in 50 mM sodium phosphate buffer (pH 7.0) containing 10 mM DTT. Subsequently, 0.025 mg/mL of each αA-crystallin variant was incubated with 0.3 mg/mL of cleaved-βB1 at 60°C in 50 mM sodium phosphate buffer (pH 7.0). Solution turbidity was monitored at 360 nm for 20 min using a V-730 spectrophotometer with continuous stirring in a temperature-regulated cell holder (JASCO, Tokyo, Japan). To confirm the chaperone-like function of the αA-crystallin variants, an insulin aggregation assay in an alternative buffer system was performed. Insulin was incubated in 20 mM Tris-HCl buffer (pH 7.8) containing 150 mM NaCl and 10 mM TCEP. Solution turbidity was monitored at 450 nm.

### Heat assay

The heat stability of each αA-crystallin variant was assessed using a V730 spectrometer (JASCO, Tokyo, Japan). Each αA-crystallin variant was prepared at a concentration of 0.2 mg/mL in 50 mM sodium phosphate buffer (pH 7.0). The temperature was incrementally raised from 20°C to 98°C at a rate of 5°C/min, as previously described [30]. The turbidity of the solution was continuously monitored at 360 nm with constant stirring throughout the experiment.

### Modification analysis of Asn151/Asp151 in the αA-crystallin variants based on LC-MS/MS

Small-scale deamidation and D/L analysis of each αA-crystallin variant were carried out following the procedures previously reported [28]. Briefly, 0.1 mg/mL of each αA-crystallin sample in 50 mM sodium phosphate buffer (pH 8.0) was aliquoted into thin-wall 8-strip PCR tubes, and 50 μL of mineral oil was added to cover the surface. The samples were heated at 50°C using a thermal cycler (MJ Research, Waltham, MA, USA). At predetermined intervals, samples were removed and stored at –20°C until further processing. For trypsin digestion, αA-crystallin samples were digested using a 50:1 protein to trypsin ratio in 50 mM Tris-HCl (pH 7.8) for 12 h at 37°C. The tryptic peptides were filtered and then introduced into a nanoscale RP-HPLC system (L-column2, 0.1 × 150 mm; CERI, Tokyo, Japan) at a flow rate of 0.5 μL/min, connected to a triple-quadrupole LC-MS 8060 mass spectrometer (Shimadzu, Kyoto, Japan). The elution was conducted over 60 min using a linear gradient from 0% to 30% of solvent A (0.1% formic acid aqueous solution) and solvent B (100% acetonitrile containing 0.1% formic acid). Multiple reaction monitoring (MRM) chromatograms for fragment ions of peptides were recorded and analyzed as total ion chromatograms in electrospray ionization positive mode with the following transitions: 437.89 > 400.20, y7 ion; 437.89 > 512.26, y4 ion; 437.89 > 375.2, y3 ion. The relative quantities of deamidation/isomerization products from Asn151 were estimated by the peak areas using Lab Solution software (Shimadzu, Japan).

### Heat unfolding assay measured by native fluorescence

To investigate the unfolding of each αA-crystallin variant induced by heat, 0.12 mg/mL of each variant was heated in 50 mM sodium phosphate buffer (pH 7.0), and fluorescence spectra were recorded using a Hitachi F-4500 spectrophotometer (Hitachi, Tokyo, Japan). The excitation wavelength was set at 295 nm, and emission spectra were recorded from 300 to 400 nm. The unfolded proteins were monitored and quantified by the fluorescence intensity ratio (337 / 373 nm) as previously documented [32]. After the heat unfolding assay, each sample was filtered through a 0.45 μm syringe filter, and the protein concentration was determined using a protein assay kit (Bio-Rad Laboratories, USA).

## Results

### Each αA-crystallin variant displayed a distinct homo-oligomeric size

**Fig 2:**
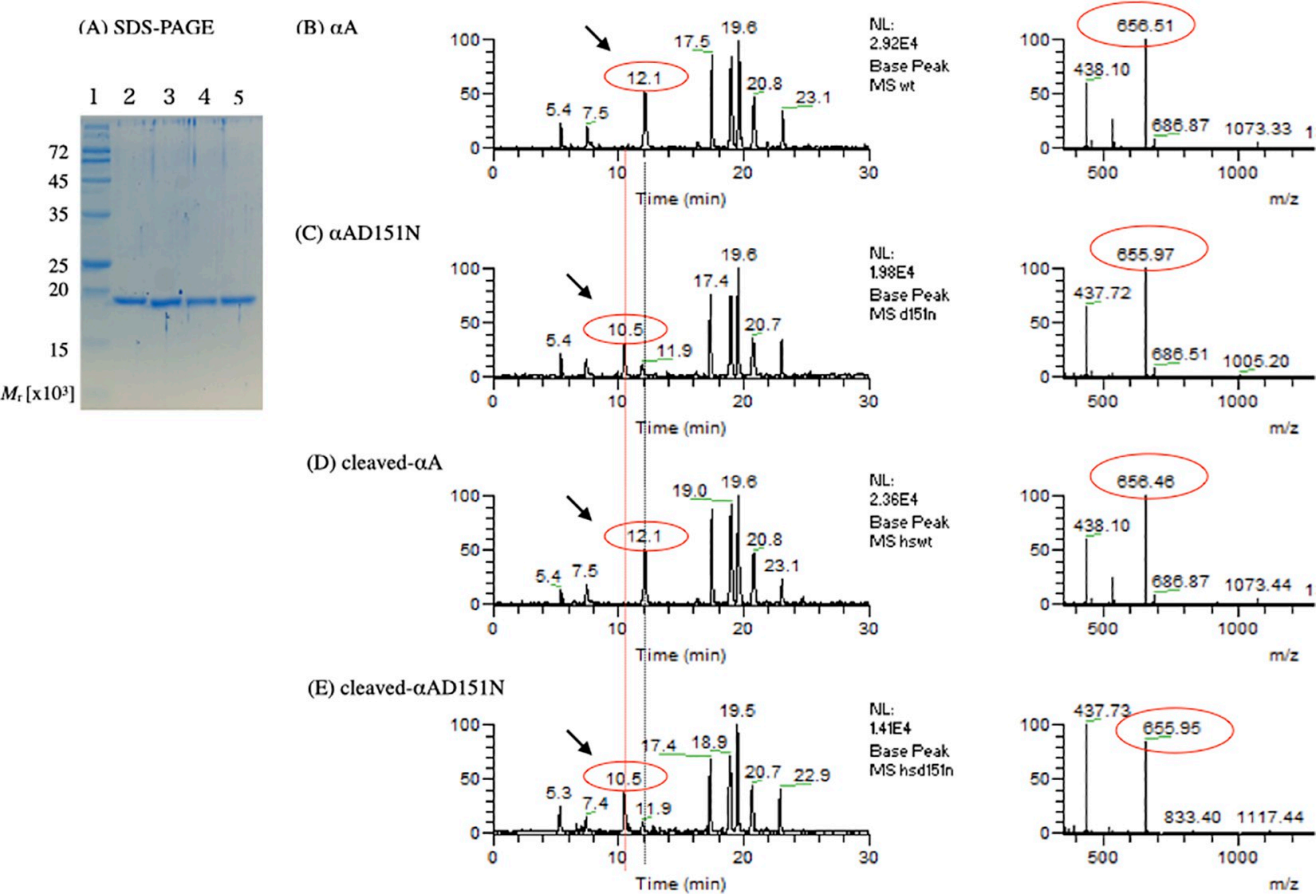
Purification and identification of αA-crystallin variants. The purity of recombinantly expressed proteins was verified by SDS-PAGE. Modifications were identified using nano-scale RP-HPLC and MS analysis for αA, αAD151N, cleaved-αA, and cleaved-αAD151N following trypsin digestion. (A) Molecular weight marker is shown on the left (lane 1). Samples are displayed as αA (lane 2), αAD151N (lane 3), cleaved-αA (lane 4), and cleaved-αAD151N (lane 5). (B–E) LC-MS chromatograms of tryptic peptides from αA, αAD151N, cleaved-αA, and cleaved-αAD151N αA-crystallin variants (left panels) and the mass of the 146–157 peptide containing Asp151 (right panels). Arrows on the left panels indicate the elution time of the 146–157 peptide. The *m*/*z* on right panels indicates the mass-to-charge ratio of each peptide. The mass-to-double charge ratio of αA peptide is 656.5.

Following the expression and purification of the αA-crystallin variants, the purity of each sample was confirmed by SDS-PAGE (Fig 2A). There was a band for his-SUMO fusion αA ~35 kDa prior to ulp-1 digestion (data not shown), while the mobility of the band decreased to be the same as that of the αA-crystallin after ulp-1 digestion (Fig 2A). All four variants (αA, αAD151N, cleaved-αA, and cleaved-αAD151N) showed a single band, consistent with the theoretical molar mass of the αA-crystallin monomeric subunit (19.9 kDa). Fig 2B–2E presents the LC chromatograms (left column) and their analyses (right column) of the αA-crystallin 146–157 peptide IQTGLD/NATHAER ([M+2H^2+^]; 656.5 / 656.0) from αA, αAD151N, cleaved-αA, and cleaved-αAD151N. The chromatograms displayed a similar elution pattern, but αAD151N and cleaved-αAD151N showed different elution times and a decrease in the molar mass of the peptide compared to αA and cleaved-αA, which aligns with the D > N mutation (Fig 2C and 2E, right column).

**Fig 3:**
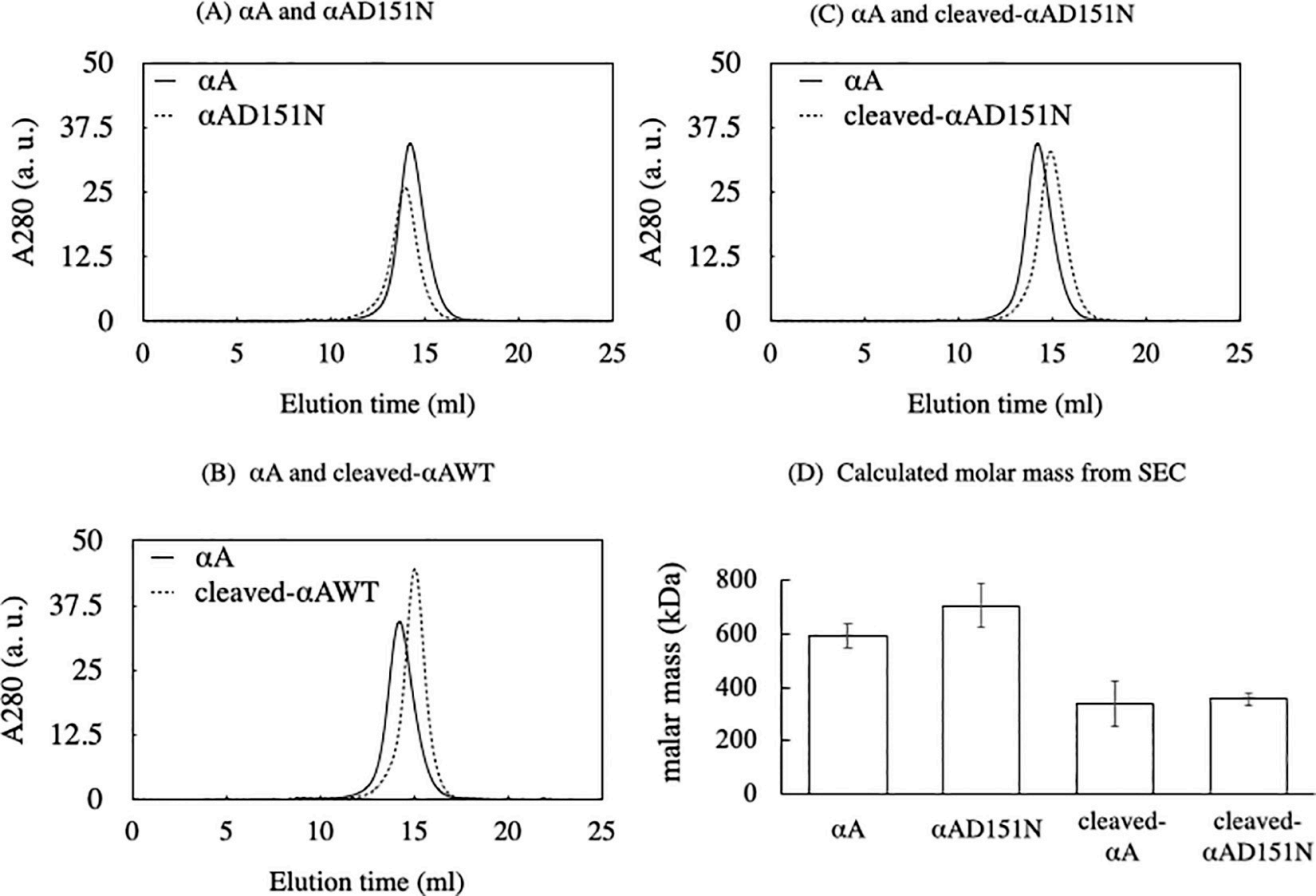
Different oligomeric sizes of αA-crystallin variants. (A–C) The size of each αA-crystallin variant was evaluated by analytical size exclusion chromatography. The black line shows the elution profile of αA. Dotted lines show the elution profiles of (A) αAD151N, (B) cleaved-αA, and (C) cleaved-αAD151N. (D) Calculated molar masses of each αA-crystallin variant, based on the elution of known standards (thyroglobulin, horse spleen ferritin, aldolase, bovine serum albumin, chicken ovalbumin, chymotrypsinogen A, and bovine pancreatic ribonuclease A). Data also show the mean ± SD from three experiments.

Each purified αA-crystallin variant underwent size-exclusion chromatography to estimate the oligomeric size (Fig 3A–3C). The calculated molar masses of αA, αAD151N, cleaved-αA, and cleaved-αAD151N were 590 ± 44 kDa, 702 ± 81 kDa, 339 ± 84 kDa, and 356 ± 22 kDa, respectively (Fig 3D and Table 1). These data indicate that each αA-crystallin variant formed different oligomeric states with varying numbers of subunits during homo-oligomerization. The number of subunits in the oligomers of cleaved-αA and cleaved-αAD151N was nearly half that of αA and αAD151N, respectively (Table 1).

**Table 1:** Calculated molar mass and predicted number of subunits from SEC.

Protein	Molar mass (kDa)	Predicted number of subunits
αA	590 ± 44	27~32
αAD151N	702 ± 81	31~40
cleaved-αA	339 ± 84	13~22
cleaved-αAD151N	356 ± 22	17~19

**Fig 4:**
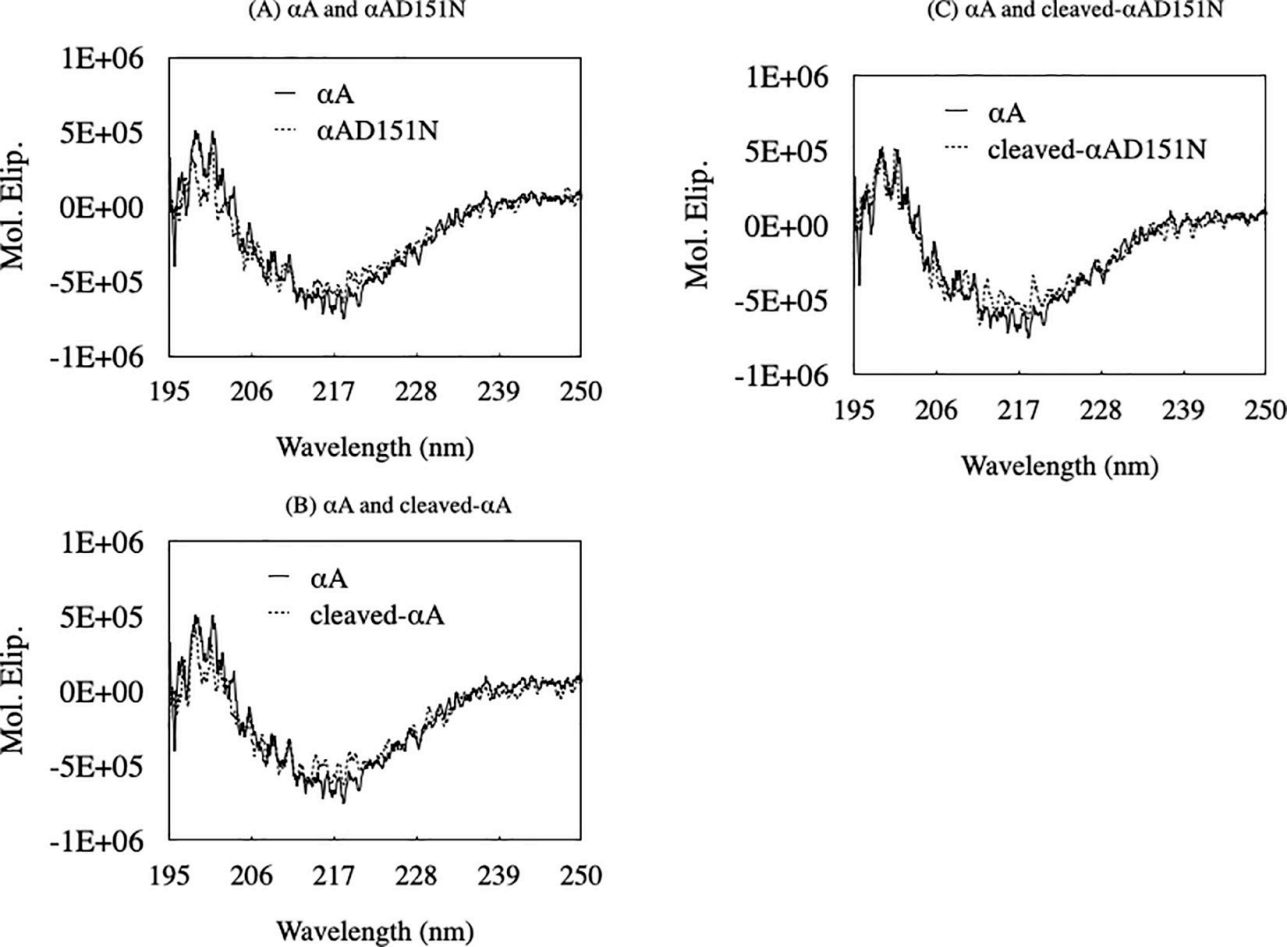
Similar secondary structure contents in αA-crystallin variants. (A–C) The secondary structure of the αA-crystallin variants was investigated by Far-UV CD. The black line shows the spectrum of αA. Dotted lines show the spectra of (A) αAD151N, (B) cleaved-αA, and (C) cleaved-αAD151N respectively. Spectra were recorded between 195 and 250 nm.

Next, to examine the secondary structure of each αA-crystallin variant, Far-UVCD spectra were obtained (Fig 4). The secondary structures of αAD151N, cleaved-αA, and cleaved-αAD151N were similar to αA (Fig 4A–4C). The Far-UVCD spectra suggested that the four αA-crystallin variants possessed nearly identical secondary structures. However, despite their similar secondary structures, the oligomeric states of the αA-crystallin variants varied significantly during homo-oligomerization based on our Far-UVCD and size-exclusion chromatography results.

### Small-size αA-crystallin variants exhibited increased hydrophobicity

**Fig 5:**
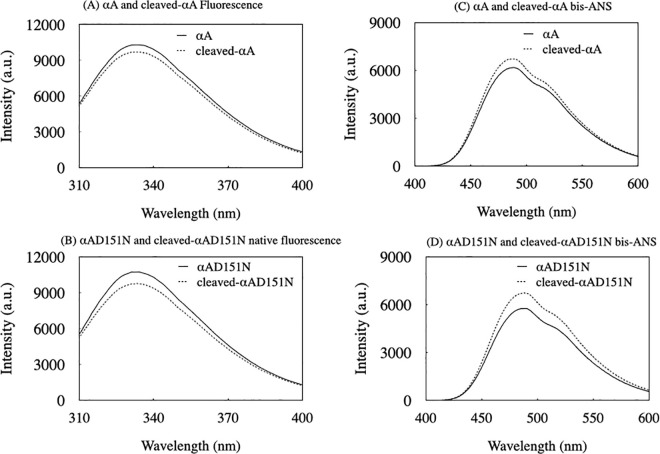
Different oligomeric states alter the native fluorescence and hydrophobicity of αA-crystallin variants. (A, B) The tertiary structure of the αA-crystallin variants was investigated by native fluorescence. The black line shows the spectrum of αA and αAD151N. Dotted lines show the spectrum of cleaved-αA, and cleaved-αAD151N. (C, D) Bis-ANS fluorescence of the αA-crystallin variants. The black line shows the spectrum of αA and αAD151N. Dotted lines show the spectrum of cleaved-αA, and cleaved-αAD151N, respectively.

There is only one tryptophan residue (Trp9) in the N-terminal extension of αA-crystallin, which was utilized to investigate the tertiary structure of the αA-crystallin variants (Fig 5A and 5B). The mutation did not significantly alter the native fluorescence spectra pattern, although the cleaved-αA-crystallin variants exhibited slightly lower Trp fluorescence intensity compared to their normal-sized counterparts. This suggests a variation in the Trp environment around the N-terminal extension of the αA-crystallin variants, with or without tag deletion. Additionally, bis-ANS, a probe used to detect the hydrophobic surface of protein tertiary structures, was applied to the αA-crystallin variants (Fig 5C and 5D). The bis-ANS fluorescence spectra indicated that cleaved-αA and cleaved-αAD151N exposed more hydrophobic surface than αA and αAD151N. These findings imply the posibility that the hydrophobic surfaces of smaller-sized αA-crystallin variants may serve as interfaces for inter-subunit interactions, forming larger oligomers, as hydrophobicity decreases with increasing size of αA-crystallin.

### The αA-crystallin variants maintained chaperone-like functions

**Fig 6:**
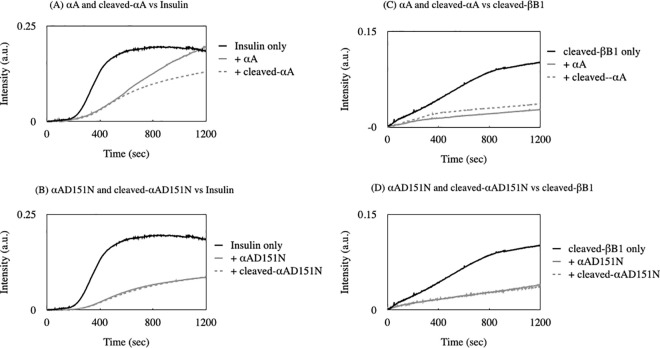
Effects of mutation on the chaperone-like function of αA-crystallin. (A, B) Insulin was incubated at 37°C in the presence or absence of αA-crystallin variants. Aggregation was initiated by adding 10 mM DTT, and turbidity was measured at 360 nm. (C, D) Recombinantly expressed cleaved-βB1 was incubated at 60°C in the presence or absence of αA-crystallin variants, and turbidity was measured at 360 nm. The black line shows the aggregation profile of the substrate. Gray solid and gray dotted lines show the aggregation profile in the presence of αA or cleaved-αA, and αAD151N or cleaved-αAD151N, respectively.

Lens αA-crystallin has chaperone-like functions that help to maintain lens transparency over a long time. To illustrate whether the mutation and change in size of αA-crystallin would impact the chaperone-like function, we tested the ability of each αA-crystallin variant to prevent DTT-induced aggregation of insulin at 37°C and heat-induced aggregation of cleaved-βB1 at 60°C (Fig 6). In the absence of αA-crystallin, insulin started to aggregate after the addition of DTT. Though αA suppressed those insulin aggregation only at the early phase, cleaved-αA effectively prevented aggregation even at the late phase of incubation (Fig 6A). The chaperone-like activity of αAD151N and cleaved-αAD151N for insulin was relatively higher than that of the αA variants (Fig 6B). The results from heat-induced aggregation of cleaved-βB1 were different from what we observed in the insulin aggregate assay. All four αA-crystallin variants suppressed cleaved-βB1 aggregates at the same level (Fig 6C and 6D). One of the important results is that the chaperone-like activity of cleaved-αA variants were more effective than that of the αA-crystallin molecules (Fig 6).

**Fig 7:**
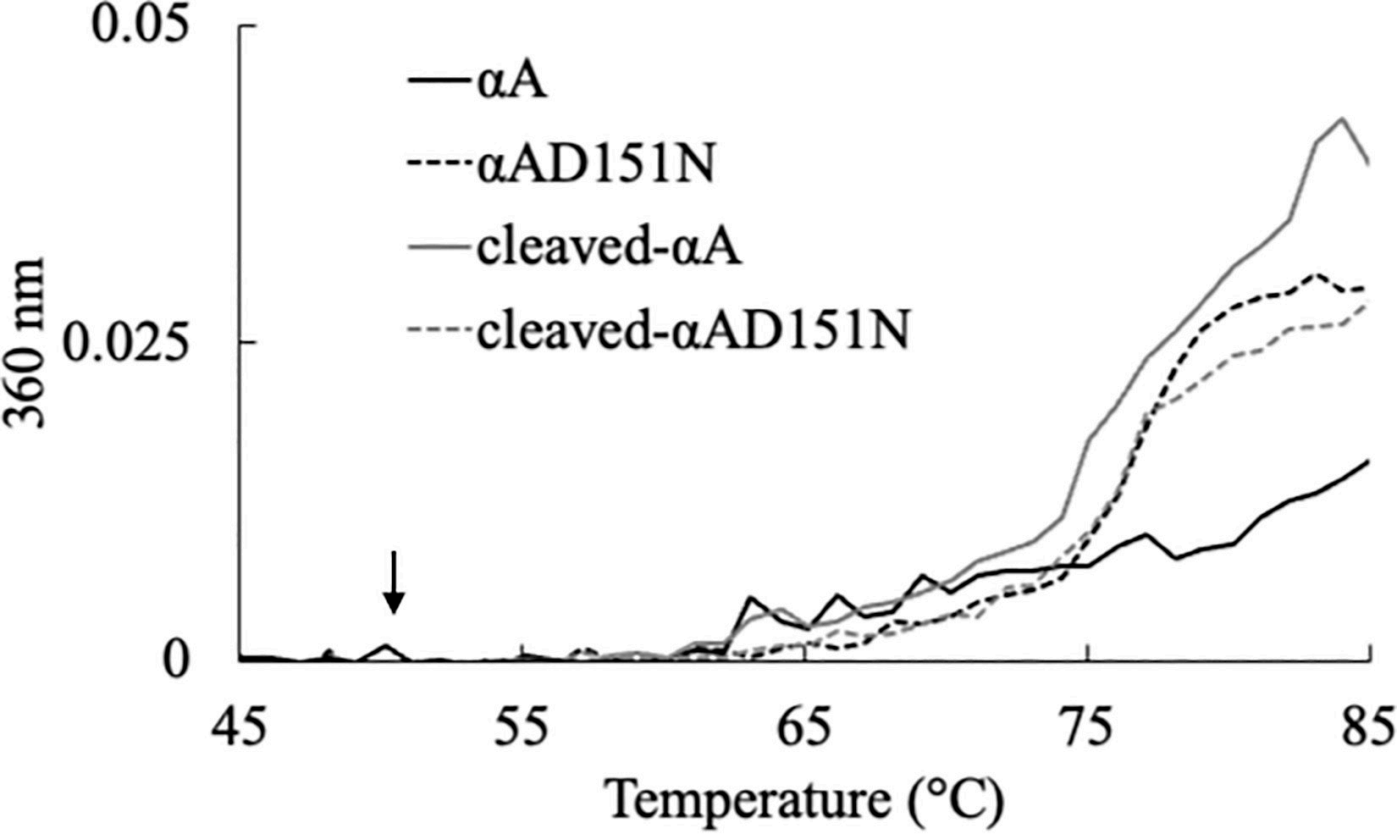
Heat stability of each αA-crystallin variant. Temperature was increased to 98°C at a rate of 5°C/min, and the heat-induced precipitation of each αA-crystallin variant was monitored at 360 nm. Black lines and dotted lines show the aggregation profile of αA and cleaved-αA variants. Grey lines and dotted lines show the aggregation profile of αAD151N and cleaved-αAD151N αA-crystallin variants. An arrow indicates the position at 50°C.

To confirm these results, another chaperone assay using a different buffer system was performed (S1 Fig). The data shows that the chaperone-like activity of αAD151N and cleaved-αAD151N was identical, while cleaved-αA was more effective than αA. These results might be due to the differences in heat stability for αA-crystallin variants, therefore the heat stability of each αA-crystallin variant was evaluated (Fig 7). The result demonstrated that solubility of all variants was similar around 50°C, but αA was the most stable one at higher temperature. αA-crystallin variants showed different heat stability, but they still could maintain their chaperone-like functions, and smaller-sized αA-crystallin variants could chaperone the substrate more effectively.

### The kinetics of deamidation/isomerization/racemization for Asp151 was specific for different states of αA-crystallin homo-oligomer

**Fig 8:**
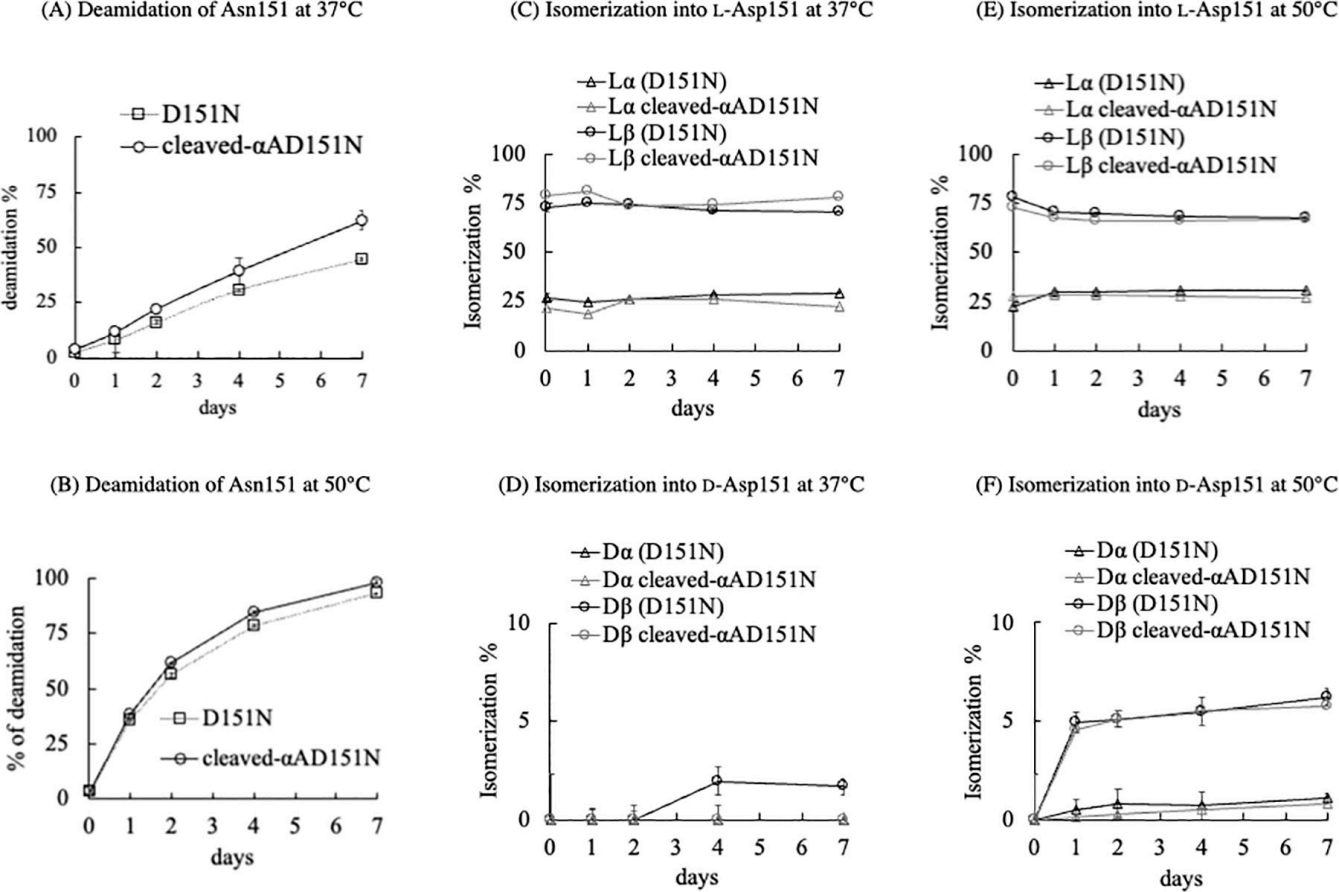
Kinetics of modifications at Asn151 of the αA-crystallin variants. (A, B) Deamidation ratio of the αAD151N variants at 37°C and 50°C. The deamidation ratio of αAD151N (open circles) and cleaved-αAD151N (open squares) increase over time. Data points represent quantification of peak areas corresponding to the deamidation of Asn151. (C, D) Isomerization ratio of the αAD151N variants at 37°C. Black and gray lines indicate the isomerization ratio to form Asp isomers in αAD151N and cleaved-αAD151N, respectively. (E, F) Isomerization ratio of the αAD151N variants at 50°C. Black and gray lines indicate the isomerization ratio to form Asp isomers in αAD151N and cleaved-αAD151N, respectively. Data points represent quantification of peak areas corresponding to the Ile146–Arg157 peptide containing L-α-Asp (closed circles), L-β-Asp (open circles), D-α-Asp (open triangles), and D-β-Asp (open squares) isomers after heating (average of three experiments). Error bars represent standard deviation from three separate experiments.

Next, we evaluated the contribution of different-sized oligomers to the deamidation/isomerization/racemization of Asp/Asn in the two αAD151N variants (Fig 8). After heating for 1 week, the αA-crystallin variants showed multiple elution peaks on the nano-scale RP-HPLC chromatograms, identified by the selected mass values of the tryptic peptides. The peak corresponding to the sequence from Ile146 to Arg157 of αA-crystallin demonstrated Asn151, or Asp151 isomerization; thus, the peak area corresponding to each Asp-containing peptide chromatogram was used to determine the yield of isomerization via deamidation [28]. We found that about 50% of Asn151 was isomerized to Asp151 via deamidation after 1 week at 37°C, and more than 85% of Asn151 was isomerized to Asp151 after 1 week at 50°C (Fig 8A and 8B). Notably, more cleaved-αAD151N deamidated than αAD151N within 1 week at both 37°C and 50°C. These differences imply that deamidation was size-dependent. By contrast, the percentage of isomerization to form L-/D-Asp was nearly the same for cleaved-αAD151N and αAD151N (Fig 8C–8F). The main isomerized product was L-β-Asp (Fig 8C and 8E). The yield of L-β-Asp at Asp151 reached 75% soon after the start of deamidation at both 37°C and 50°C, but then remained at the same level during incubation. Notably, right after the incubation at 37°C started, slightly more isomerization was seen in cleaved-αAD151N than in αAD151N. On the other hand, a small amount of racemized products formed after 1 week of incubation (Fig 8D, 8F, and S2 Fig): 2.5% of D-β-Asp at 37°C and 5.5% at 50°C. The amount of D-α-Asp formed under 37°C and 50°C incubation was less than 1% (Fig 8D, 8F, and S2 Fig). Incubation of αA-crystallin variants at 50°C for 7 days may induce protein aggregation and degradation. These modifications can impact the isomerization ratio of each αA-crystallin variant. The extent of modifications after heating at 50°C for 1 week was analyzed by 12% SDS-PAGE (S3 Fig), showing some bands of αA-crystallin higher-order oligomers. Comparatively, the majority of αA-crystallin variants remained unmodified. Therefore, while aggregation and degradation of αA-crystallin variants may influence the isomerization rate at Asn151, their impact is not considered significant enough to affect the results.

### Isomerization of Asp151 did not induce precipitation of αA-crystallin

**Fig 9:**
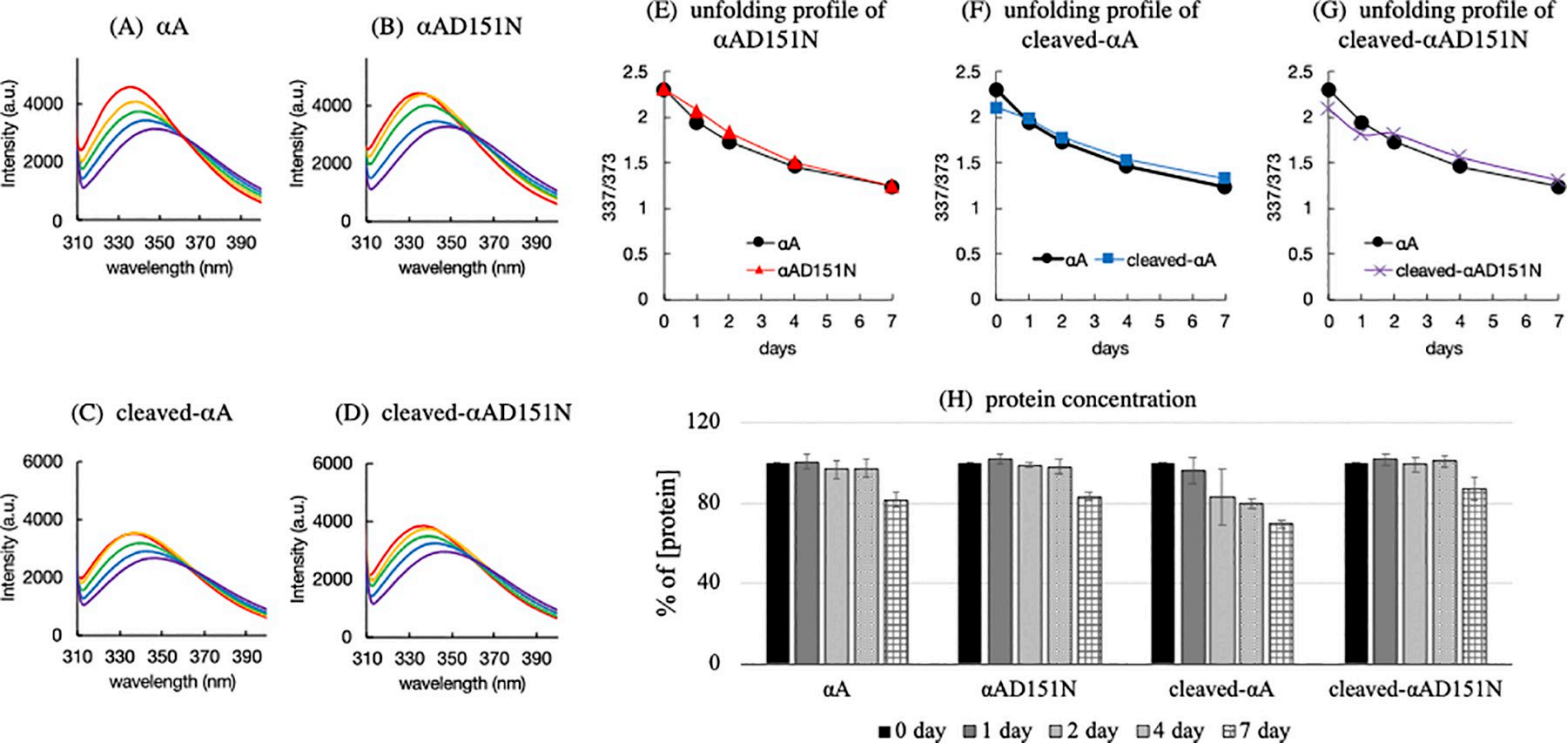
Heat-induced unfolding of the αA-crystallin variants. The environment and estimated tertiary structural alterations around Trp9 in the N-terminal extension of αA-crystallin variants were probed by monitoring the fluorescence maxima at 337 / 373 nm during incubation for 7 days at 50°C. Colored lines indicate the fluorescence profile of Trp9 in (A) αA, (B) αAD151N, (C) cleaved-αA, and (D) cleaved-αAD151N αA-crystallin after 0 (red), 1 (orange), 2 (yellow), 4 (blue), and 7 (purple) days of incubation. Unfolding profiles of (E) αAD151N, (F) cleaved-αA, and (G) cleaved-αAD151N αA-crystallin were calculated from each ratio of fluorescence maxima at 337 / 373 nm during incubation. (H) Protein concentration during the unfolding assay. Error bars represent standard deviation from three separate experiments.

To observe the change in solubility of αA-crystallin after isomerization, the folding state and solubility of the αA-crystallin variants were evaluated in a heat assay by monitoring the native fluorescence and protein concentration at various time points (Fig 9). Fig 9A indicated the unfolding of αA during incubation at 50°C for 1 week. The peak intensity at 337 nm decreases and the curve shows a small shift to a higher wavelength with an increase in the peak intensity at 373 nm, indicating the partial unfolding of αA variants. The αA-crystallin variants showed a similar unfolding pattern to that of αA (Fig 9B–9D). To further compare the unfolding state of each αA-crystallin variant, the relative fluorescence intensity calculated by 337 / 373 nm was compared at each time point. The result suggests that the variants had similar heat stability under the experimental conditions of the heat assay (Fig 9E–9G). Under constant heating for days, isomerization happened with the unfolding process of αA-crystallin. The unfolding process might cause the formation of insoluble aggregates, but over 80% of our cleaved-αAD151N and αAD151N variants remained soluble after 1 week of incubation under heat, indicating that isomerization did not impact the solubility of αA-crystallins (Fig 8H). By contrast, cleaved-αA tended to lose its soluble proteins after 1 week.

## Discussion

Cleaved-αA and cleaved-αAD151N were smaller in oligomeric state than their corresponding normal-sized αA and αAD151N (Fig 3). The sumo protein tag on the N-terminal region of αA-crystallin, which is similar in size to the αA-crystallin subunit, is likely to disturb subunit–subunit interactions in protein expression and/or oligomerization steps [4, 29]. The four variants may have different subunit structure due to tag expression. However, trypsin digestion efficiency and CD data for all variants showed a highly similar pattern (Fig 2B–2E left panels and Fig 4). These results implied that the N-terminal tag did not greatly alter the monomeric structure of αA-crystallin.

Asp151 is located in the relatively flexible region on the C-terminus, and this may be why the mutation of D>N does not alter the secondary structure of αA-crystallin much (PDB 3L1F) [33]. In a recent study, a polydisperse model of αA-crystallin was generated, reflecting the multiple interfaces at the C-terminal domain of four αA-crystallin subunits formed by hydrogen bonding, inter-subunit and intra-subunit interactions (PDB 6T1R) [6]. In this model, Asp151 participates in the intra-subunit interaction with Ser121, and this intra-subunit interaction may compete with other inter-subunit interactions at the C-terminus of αA-crystallins. Our data did not show a difference in structures among αA-crystallin variants. Probably, the contribution of this D > N variant at the C-terminal flexible region to the tertiary structure is minimal.

All of the αA-crystallin variants demonstrated chaperone-like functions, effectively preventing the aggregation of both insulin and cleaved-βB1 (Fig 6 and S1 Fig). Specifically, the chaperone-like functions of cleaved-αA, cleaved-αAD151N, and αAD151N in preventing insulin aggregation were more effective than those of αA, especially during the later phases of incubation (Fig 6A, 6B, and S1 Fig). This suggests that smaller αA-crystallin oligomers interact more effectively with the insulin beta-chain, thereby inhibiting further aggregation. It has been reported that partial perturbation of αA-crystallin increases hydrophobicity and enhances its chaperone-like function [34, 35]. Our findings further substantiate this observation, showing that the smaller-sized αA-crystallin homomers, specifically cleaved-αA and cleaved-αAD151N, were more hydrophobic than the normal-sized αA-crystallin homomers (Fig 5C and 5D).

The differences in chaperone-like activity among the αA-crystallin variants for cleaved-βB1 were minimal, indicating a common mechanism preventing aggregation across all variants (Fig 6C and 6D). Previous research has highlighted the significance of the negative charge at Asp151 in the chaperone-like function of αA-crystallin, particularly in suppressing βL-crystallin aggregation [30]. This suggests that the negative charge at Asp151 may be crucial for interacting with some members of the β-crystallin family, although it appears not to be as critical for βB1-crystallin. Overall, our findings indicate that interactions between αA-crystallin and each substrate are specific.

While no significant alterations were observed in the local structure of αA-crystallin with or without tags during purification, the presence of an additional tag on the N-terminal of αA-crystallin might affect its chemical properties. Further investigation is required to explore these effects. Asn151 is prone to isomerization to form Asp when heated, and it is necessary to understand how this isomerization affects the solubility of αA-crystallin under such conditions. Our results suggest that deamidation/isomerization/racemization at position 151 of the αA-crystallin sequence does not significantly impact the solubility of αA-crystallin during heating at 50°C, as previously proposed (Fig 7) [30].

To assess the impact of differences in tertiary structure on deamidation/isomerization/racemization, we compared the modification kinetics between αAD151N and cleaved-αAD151N (Fig 8). The D/L analysis indicated that deamidation occurred to a lesser extent in αAD151N than in cleaved-αAD151N at both 37°C and 50°C, suggesting that the smaller-sized αA-crystallin homomer is more conducive to the deamidation of Asn151 (Fig 8). The initiation of deamidation involves an attack by the lone electron pair on the nitrogen of Asp151 on the adjacent carbonyl group (Fig 1). This early phase may be influenced by the steric effects associated with larger homo-oligomerization of αA-crystallin (Table 1 and Fig 8A). An increase in isomerization was observed in cleaved-αAD151N both at the early and late phases of incubation, suggesting changes in the equilibrium of isomerization (Fig 8C–8F). This phenomenon could be due to heat-induced further oligomerization of cleaved-αAD151N or partial unfolding of the protein enhancing isomerization. In conclusion, our findings demonstrate that smaller-sized αA-crystallin homomers facilitate isomerization at Asp/Asn151, whereas the normal-sized αA-crystallin homomers exhibit less isomerization, possibly due to greater steric hindrance around Asp/Asn151.

The simultaneous isomerization and racemization of Asp151 increase in the water-insoluble fraction of aged lens [15], thus, these modifications are predicted to decrease the heat stability of αA-crystallin *in vivo*. Fig 9 suggests that although αA and cleaved-αA possessed similar unfolding profiles, cleaved-αA exhibited less heat stability than αA (Figs 7 and 9H).

Heat stability of all αA-crystallin variants was not altered below 65°C *in vitro*, suggesting that heat stability was not affected by the percentage of isomerization differences among all variants *in vitro* (Fig 7). Notably, the isomerization/racemization of Asp151 to D-β-Asp151 in αA-crystallin *in vivo* starts shortly after birth, and the ratio is very high [15, 17, 30]. However, our results predominantly showed the formation of L-β-Asp151. This suggests the presence of specific factors *in vivo* that enable the formation of enol-type reaction intermediates, producing only D-Asp at an early life stage.

## Conclusion

Our present results indicate that the size of the αA-crystallin tertiary structure impacts the initial event for both modifications. Additionally, our results imply the presence of specific factors that likely influence the isomerization of Asp residues *in vivo*. To clarify the mechanism, we developed a model combining the mutation of Asp151 to Asn151, which accelerates the isomerization at position 151 of αA-crystallin, with a cleaved-tag purification system that produces different-sized αA-crystallin homo-oligomers. This model serves as a useful tool for screening factors that affect αA-crystallin stability and isomerization.

## Supporting information

S1 Fig Impact of αA-crystallin variants on the chaperone-like function for TCEP-induced aggregation of insulin in tris buffer systems. (A) αA-crystallin variants ranging from 0.1–1.0 mg/mL were incubated with 0.4 mg/mL of insulin in 50 mM Tris-HCl buffer (pH 7.8) containing 150 mM NaCl and 10 mM TCEP. Turbidity of the solution was monitored at 450 nm for 30 minutes using a V730 spectrophotometer with a temperature-regulated cell holder and constant stirring (JASCO, Tokyo, Japan). The bold black line represents the aggregation profile of the insulin. Black solid, black dotted, and gray solid lines represent the aggregation profile in the presence of 0.1 mg/mL, 0.4 mg/mL, and 1.0 mg/mL of αA, respectively. (B) 1.0 mg/mL of each αA-crystallin variant was incubated with 0.4 mg/mL of insulin in 20 mM Tris-HCl buffer containing 150 mM NaCl and 10 mM TCEP. The bold black line indicates the aggregation profile of the insulin. Black solid, black dotted, gray solid, and gray dotted lines represent the aggregation profile in the presence of αA, cleaved-αA, αAD151N, and cleaved-αAD151N, respectively.
(TIFF)

S2 Fig LC-MS-MRM chromatograms of the Asp/Asn151 containing peptide from cleaved-αAD151N after 1 week of incubation at 37°C. D/L analysis using the LC-MS-MRM system was performed. Cleaved-αAD151N was heated at 37°C for 7 days, then digested by trypsin using conventional methods. The elution order of each Asp/Asn-containing peptide at this site was Asn > D-α-Asp > L-α-Asp > L-β-Asp > D-β-Asp. All elution profiles were identified using a synthetic peptide loaded on the same systems. Details of the analysis parameters are described under the chromatograms.
(TIFF)

S3 Fig Heat incubation induced a small amount of aggregate/degradation of αA-crystallin variants. A-crystallin variants before and after heat incubation were analyzed using 12% reducing SDS-PAGE. M indicates molecular weight markers. Numbers above each lane indicate the incubation temperature. The bold triangle indicates the original size of αA, cleaved-αA, αAD151N, or cleaved-αAD151N. All samples were incubated for 4 days (A) or 7 days (B) at the same concentration (1.3 mg/mL) in 50 mM Na-phosphate buffer (pH 8.0) and loaded in the same amount for each lane.
(TIFF)

S1 Raw image (PDF)
