# Supplementary figures and images for: Characterization of different-sized human αA-crystallin homomers and implications to Asp151 isomerization
Source: PLoS One. 2024 Jul 11;19(7):e0306856. doi: 10.1371/journal.pone.0306856 (PMC11238991; doi:10.1371/journal.pone.0306856)

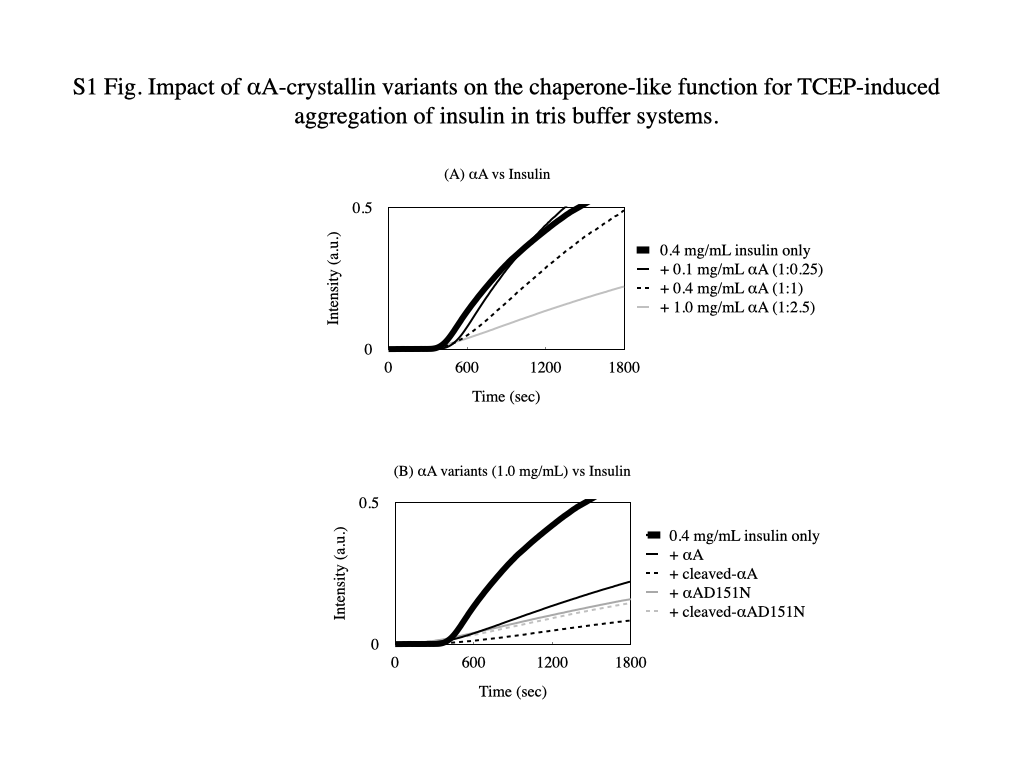

Supplement: S1 Fig — (A) αA-crystallin variants ranging from 0.1–1.0 mg/mL were incubated with 0.4 mg/mL of insulin in 50 mM Tris-HCl buffer (pH 7.8) containing 150 mM NaCl and 10 mM TCEP. Turbidity of the solution was monitored at 450 nm for 30 minutes using a V730 spectrophotometer with a temperature-regulated cell holder and constant stirring (JASCO, Tokyo, Japan). The bold black line represents the aggregation profile of the insulin. Black solid, black dotted, and gray solid lines represent the aggregation profile in the presence of 0.1 mg/mL, 0.4 mg/mL, and 1.0 mg/mL of αA, respectively. (B) 1.0 mg/mL of each αA-crystallin variant was incubated with 0.4 mg/mL of insulin in 20 mM Tris-HCl buffer containing 150 mM NaCl and 10 mM TCEP. The bold black line indicates the aggregation profile of the insulin. Black solid, black dotted, gray solid, and gray dotted lines represent the aggregation profile in the presence of αA, cleaved-αA, αAD151N, and cleaved-αAD151N, respectively. (TIFF) [file pone.0306856.s001.tiff]

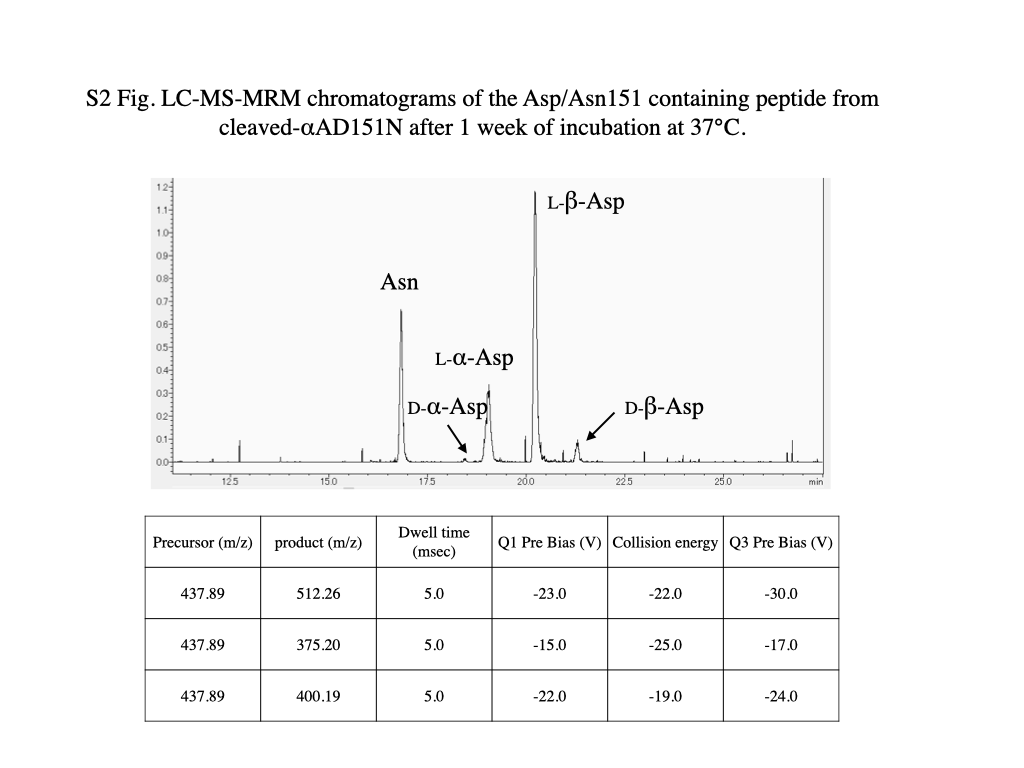

Supplement: S2 Fig — D/L analysis using the LC-MS-MRM system was performed. Cleaved-αAD151N was heated at 37°C for 7 days, then digested by trypsin using conventional methods. The elution order of each Asp/Asn-containing peptide at this site was Asn > D-α-Asp > L-α-Asp > L-β-Asp > D-β-Asp. All elution profiles were identified using a synthetic peptide loaded on the same systems. Details of the analysis parameters are described under the chromatograms. (TIFF) [file pone.0306856.s002.tiff]

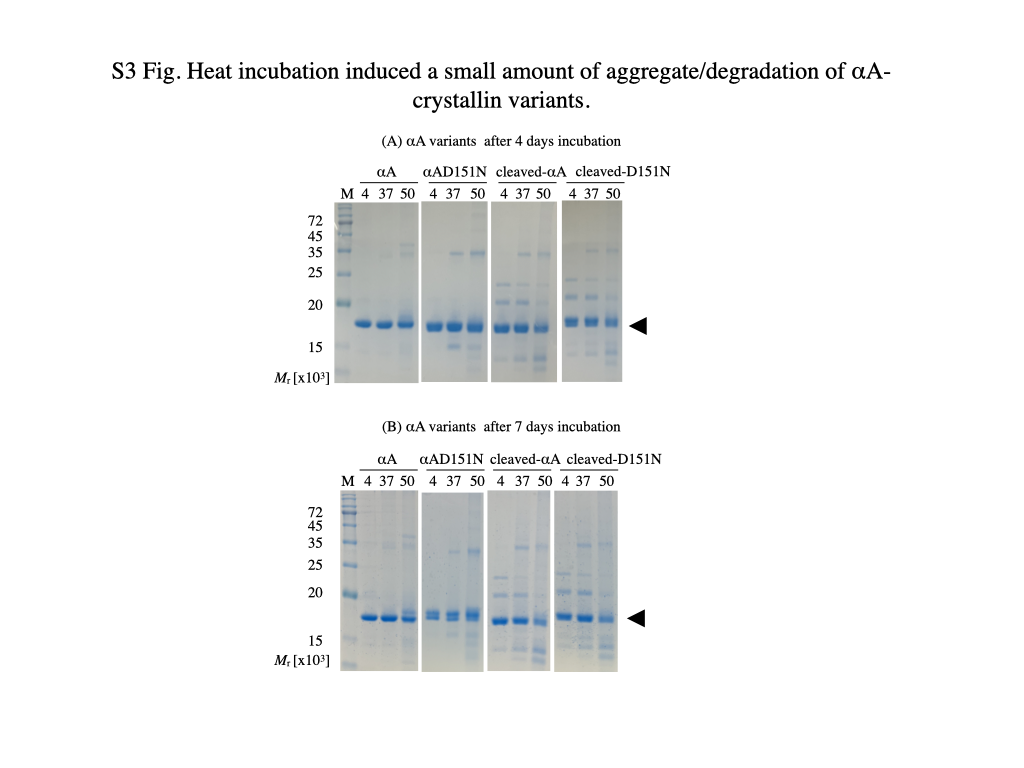

Supplement: S3 Fig — A-crystallin variants before and after heat incubation were analyzed using 12% reducing SDS-PAGE. M indicates molecular weight markers. Numbers above each lane indicate the incubation temperature. The bold triangle indicates the original size of αA, cleaved-αA, αAD151N, or cleaved-αAD151N. All samples were incubated for 4 days (A) or 7 days (B) at the same concentration (1.3 mg/mL) in 50 mM Na-phosphate buffer (pH 8.0) and loaded in the same amount for each lane. (TIFF) [file pone.0306856.s003.tiff]
