# Supplementary material for: Characterization of different-sized human αA-crystallin homomers and implications to Asp151 isomerization
Source: PLoS One. 2024 Jul 11;19(7):e0306856. doi: 10.1371/journal.pone.0306856 (PMC11238991; doi:10.1371/journal.pone.0306856)

Fig 2. Purification and identification of αA-crystallin variants.

(A) SDS-PAGE

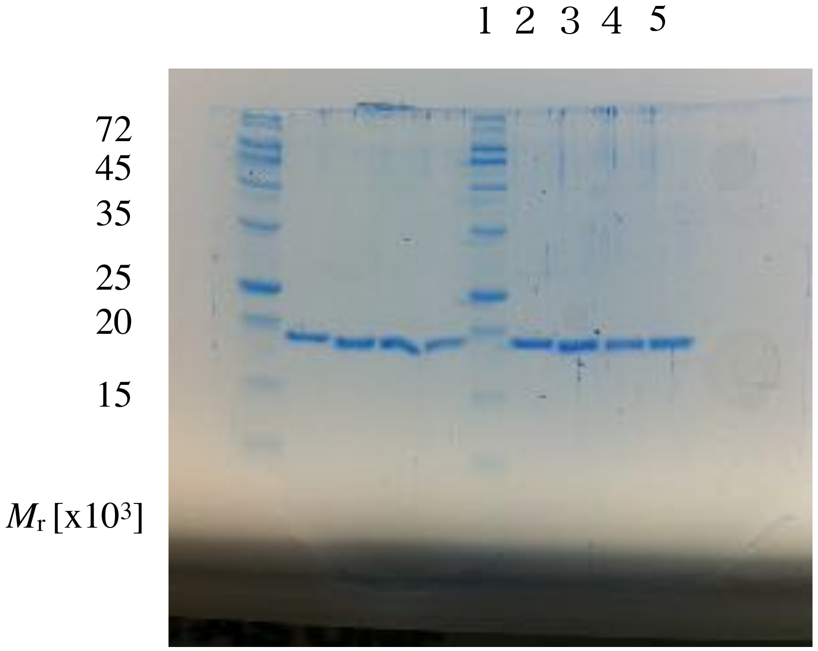

S3 Fig. Heat incubation induced a small amount of aggregate/degradation of αA-crystallin variants.

(A) αA variants after 4 days incubation

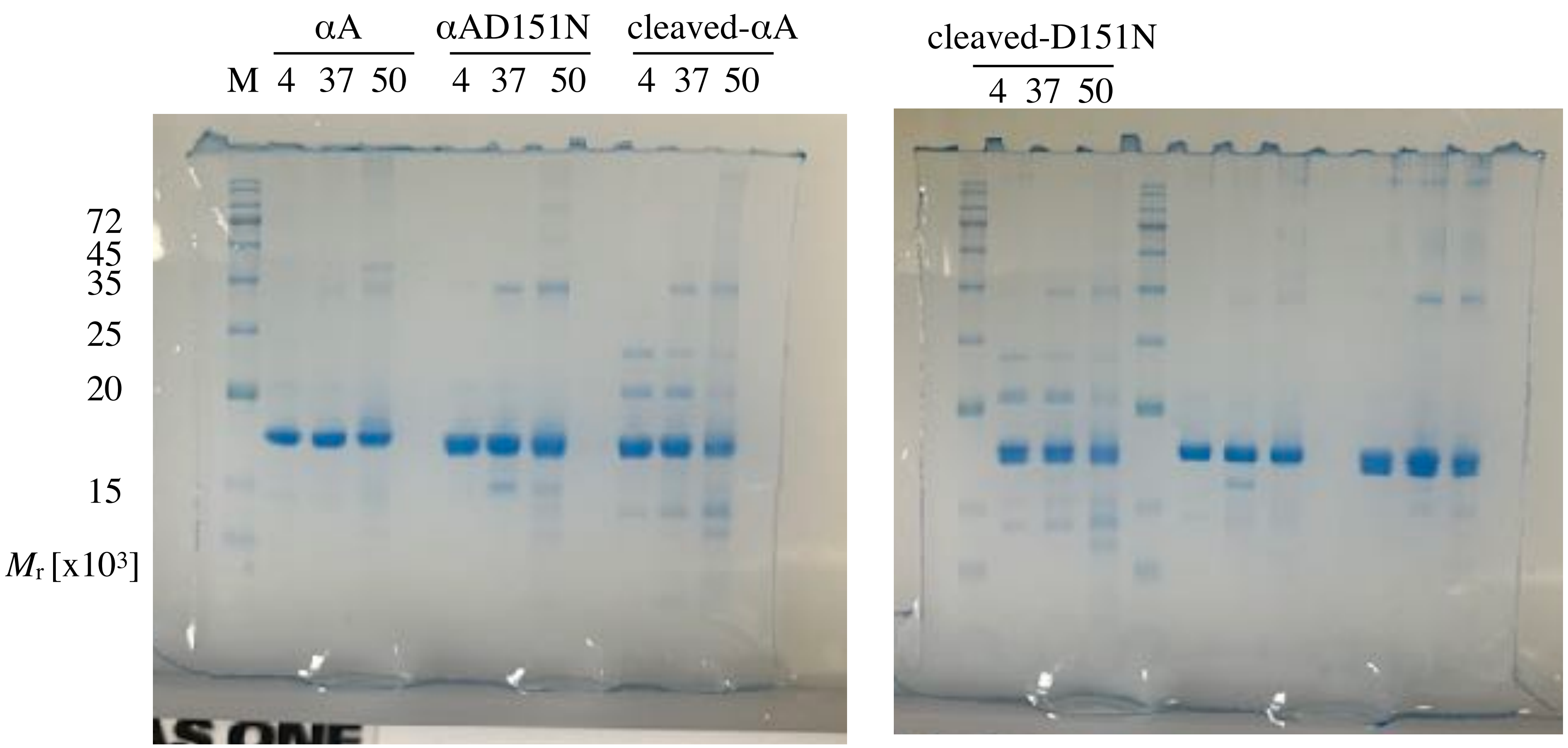

(B) αA variants after 7 days incubation

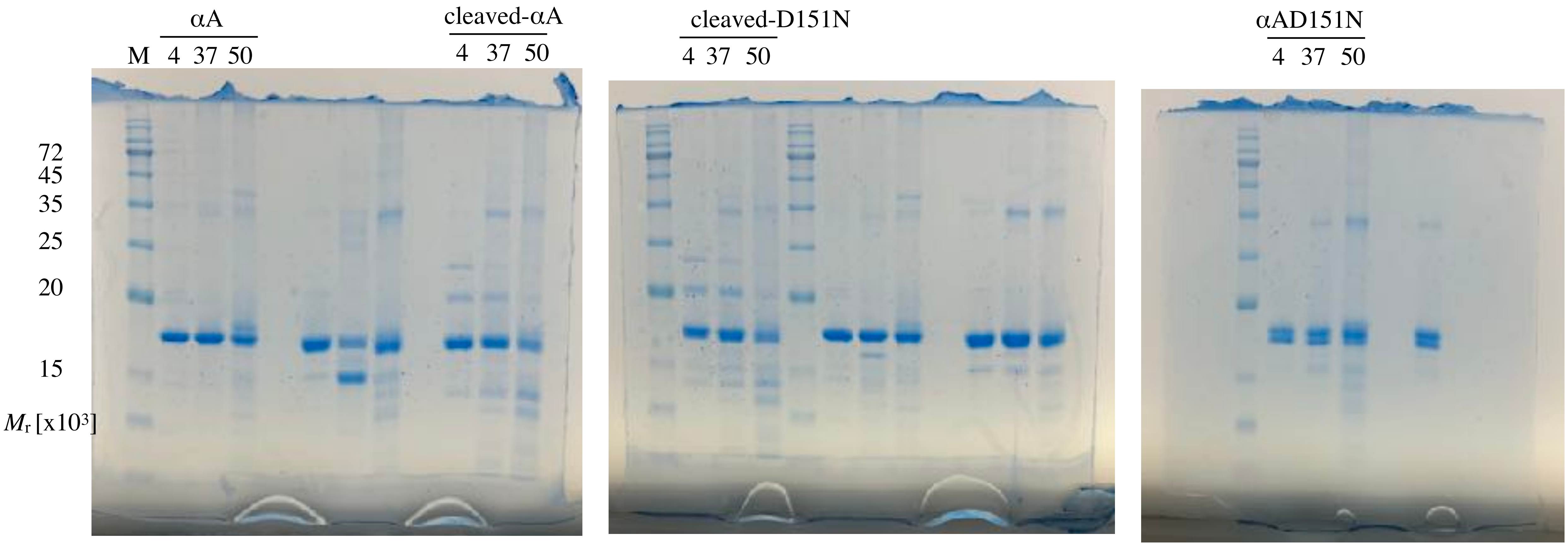

Supplement: S1 Raw image — (PDF) [file pone.0306856.s004.pdf]
